# Surveillance on schistosomiasis in five provincial-level administrative divisions of the People’s Republic of China in the post-elimination era

**DOI:** 10.1186/s40249-020-00758-4

**Published:** 2020-10-01

**Authors:** Jing-Yi Guo, Jing Xu, Li-Juan Zhang, Shan Lv, Chun-Li Cao, Shi-Zhu Li, Xiao-Nong Zhou

**Affiliations:** National Institute of Parasitic Diseases, Chinese Center for Disease Control and Prevention, WHO Collaborating Centre for Tropical Diseases, Chinese Center for Tropical Disease Research, Shanghai, 200025 People’s Republic of China

**Keywords:** Schistosomiasis, China, Elimination, Surveillance, Evaluation

## Abstract

**Background:**

The People’s Republic of China (P. R. China) has made significant progress on schistosomiasis control. Among the 12 provincial-level administrative divisions (PLADs) with schistosomiasis endemic in P. R. China, Guangdong, Shanghai, Fujian, Guangxi and Zhejiang PLADs (following as five PLADs) had successively eliminated schistosomiasis during 1985–1995. However, consolidation of the schistosomiasis elimination in these five PLADs remains challenging. In the current study, we sought to understand the epidemic situation in these post-elimination areas and their surveillance capabilities on schistosomiasis.

**Methods:**

Annual data reflecting the interventions and surveillance on human beings, cattle and snails based on county level from 2005 to 2016 were collected through the national schistosomiasis reporting system and the data were analyzed to understand the epidemic status of schistosomiasis in the five PLADs. A standardized score sheet was designed to assess the surveillance capacity for schistosomiasis of selected disease control agencies in five PLADs and ten counties. Assessment on surveillance capacity including schistosomiasis diagnostic skills, identification of snails’ living and infection status and knowledge about schistosomiasis and its control were made. Descriptive analysis was used to analyze the epidemic status and evaluation results on surveillance capacities.

**Results:**

The assessments showed that no local cases in humans and cattle or infected snail were found in these five PLADs since 2005. However, from 2005 to 2016, a total of 221 imported cases were detected in Zhejiang, Shanghai and Fujian, and 11.98 hm^2^ of new snail habitats were found in Zhejiang, Shanghai and Guangxi. In addition, snail infestation reoccurred in 247.55 hm^2^ of former snail habitats since 2011. For the surveillance capacity assessment, the accuracy rate of IHA and MHT were 100 and 89.3%, respectively. All participants could judge the living status of snails accurately and 98.1% on the infection status of snails. The accuracy rate of the questionnaire survey was 98.0%.

**Conclusions:**

Elimination of schistosomiasis was consolidated successfully in five PLADs of P. R. China due to effective and strong post-elimination surveillance. Comprehensive consolidation strategies should be focused on the elimination of residual snails and the prevention of imported infection sources to consolidate the achievements of schistosomiasis control.

## Background

Schistosomiasis, caused by parasitic trematode blood-dwelling flukes called *Schistosoma*, is one of the most important neglected tropical diseases in the world in terms of public health impacts [1, 2]. According to the report of World Health Organization (WHO), schistosomiasis is transmitted in 78 countries around the world. In 2017, at least 220.8 million people needed prophylactic treatment for schistosomiasis [3]. In the past decade, with the increased quantity of donation of praziquantel by international organizations and companies and increased willingness to give priority for schistosomiasis control or elimination by governments of endemic countries, great progress had been obtained in many countries. Meanwhile, elimination of schistosomiasis is regarded as an achievable goal in endemic regions or countries if continuous interventions and adequate resources are provided.

Schistosomiasis japonica is the only disease caused by schistosomes for human beings and livestock in P. R. China, distributed in 12 provinces along and south of Yangtze River with a long history. Investigations conducted in the 1950s proved that there were 380 endemic counties within 12 provinces, with about 12 million people and 1.2 million cattle infected with schistosomes, and over 100 million people at risk of infection. In addition, the total habitat area of *Oncomelania hupensis*, the only intermediate host of *S. japonicum*, was approximately 14.5 billion m^2^ [4–7]. Following three decades of unremitting efforts with control strategies shifted from snails control to morbidity control, Guangdong (1985), Shanghai (1985), Fujian (1987), Guangxi (1989) and Zhejiang (1995) eliminated schistosomiasis successively.

Being a zoonotic parasitic disease, the transmission of schistosomiasis japonica is influenced by biological, natural and social factors. Multiple studies proved that schistosomiasis easily rebounded or spread to new areas due to weakened interventions, ecological changes caused by flooding, construction of water conservancy projects, increased migration of goods or human resources etc., without a sensitive surveillance and response system [8–11]. As Shanghai, Guangdong, Fujian, Guangxi and Zhejiang had eliminated schistosomiasis at least 20 years before, we evaluated the epidemic situation and the surveillance capabilities on schistosomiasis among the five PLADs, to facilitate the consolidation of elimination achievements in post elimination era and provide reference for other regions where schistosomiasis had been eliminated or will be eliminated.

## Methods

### Study sites and research design

**Fig. 1:**
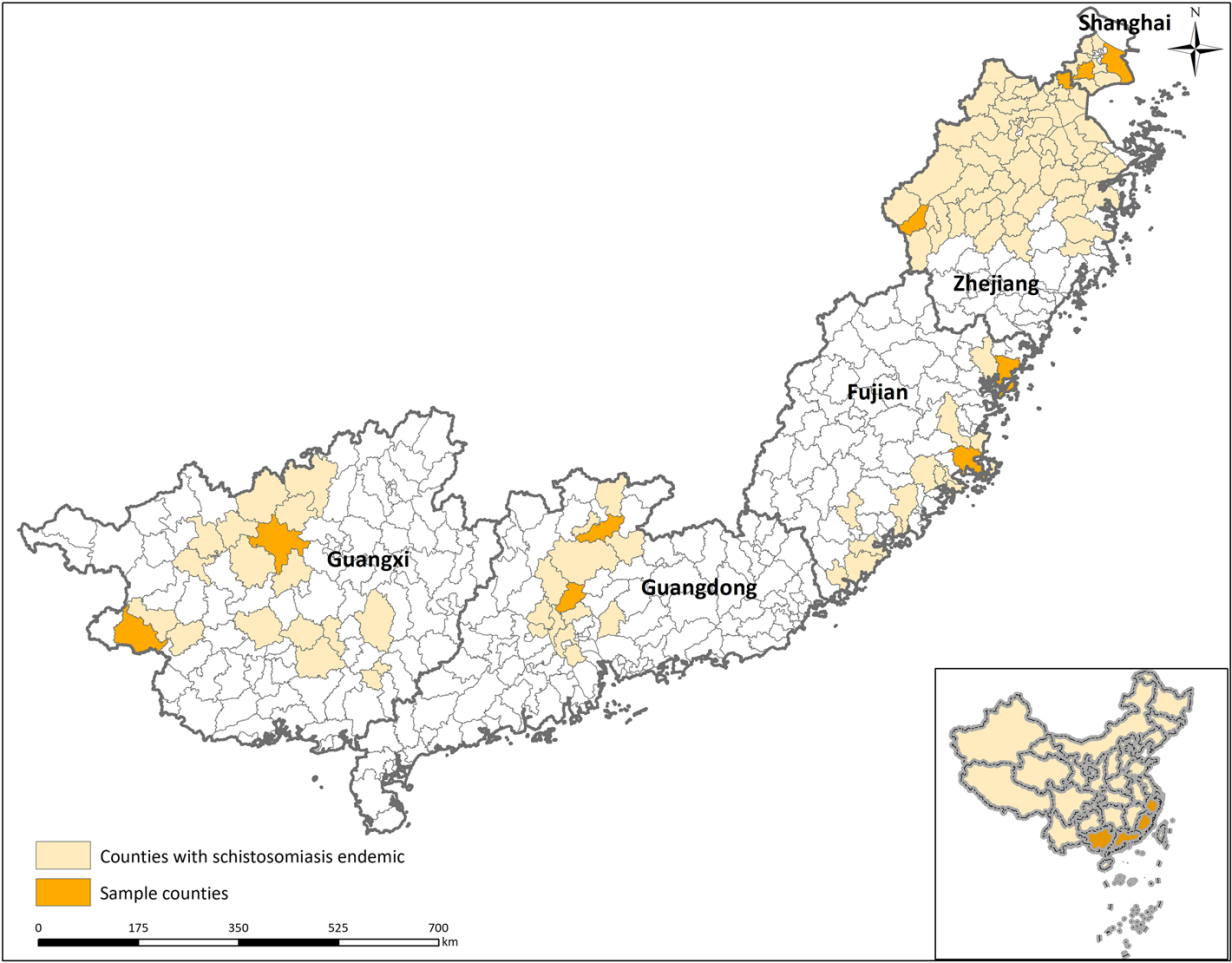
The location of research settings

Among the five PLADs, Shanghai and Zhejiang are located in the Yangtze River Delta, in the east of China, Fujian, Guangdong and Guangxi are located in the south of China. According to the epidemiological characteristics, the endemic areas in Shanghai belong to waterway-network regions, while the endemic areas in Zhejiang, Guangdong, Guangxi and Fujian are mainly hilly and mountainous regions (Fig. 1).

The study consisted of two parts: (i) Data reflecting the schistosomiasis intervention and surveillance conducted in Shanghai, Guangdong, Fujian, Guangxi and Zhejiang PLADs based on county level were collected and analyzed to understand the epidemic status of schistosomiasis. (ii) A standardized score sheet was designed to assess the surveillance capacity for schistosomiasis of selected disease control agencies.

### Retrospective data collections

A comprehensive surveillance strategy focused on clearing the internal snail habitats and infection source, preventing imported snails and cases from other provinces with ongoing schistosomiasis transmission was conducted in the five PLADs. Annual data reflecting the interventions and surveillance on human beings, cattle and snails based on county level from 2005 to 2016 were collected through the national schistosomiasis reporting system. Variables could be split into three categories: variables reflecting serological tests and stool examination for schistosomiasis (people with serum positive would do stool examinations if available and some floating people with higher infection risk from other epidemic provinces may get stool examination directly) on humans; variables on serological tests and stool examinations for schistosomiasis on cattle, and variables reflecting the distribution of *Oncomelania* snails including total habitats, new infested areas, etc.

### Assessment on surveillance capacity

A standardized score sheet was developed to assess the surveillance capacity after consulting experienced experts in the field of schistosomiasis control. The score sheet included two parts: the first part focused on the capacity of testing skills including schistosomiasis diagnostic skills, identification of snails’ living and infection status among the professionals; the second part was to assess the knowledge level about schistosomiasis and its control among the professionals. The assessment was implemented during December 2015–March 2016 in a blind manner.

#### Assessment on schistosomiasis testing skills

Laboratory testing skills of schistosomiasis were performed at provincial and county level among laboratory professionals. Two counties were selected in each province. The sample counties were selected according to their endemic situation before elimination. Counties with more schistosomiasis patients or higher density of snails were selected to assess their surveillance capacity. The assessed testing skills included: (i) Diagnostic skills included the indirect hameagglutination assay (IHA) and the miracidia hatching technique (MHT), which are most widely used in low endemic areas with light infection intensity for population screening and confirmation of schistosome infection respectively. (ii) Snails dissection and microscopic method to identify the snails’ living and infection status.

##### Preparation of reference panels for assessment

To ensure the consistence and comparability of the assessed results among different provinces, reference panels for testing methods were prepared and coded by National Institute of Parasitic Diseases, Chinese Center for Disease Prevention and Control (NIDP, China CDC). Each panel for IHA contained five serum samples (four from schistosomiasis cases and one from healthy persons), while panels for MHT included two samples containing mature eggs of schistosome obtained from the liver of infected rabbits and three negative samples with boiled-water treated eggs. Each snail panel consisted of three dead *Oncomelania* snails and seven living snails, while three of living snails were infected by schistosomes confirmed by shedding method. The test results were determined by technicians from NIPD, China CDC and were regarded as gold standard. The same patch of diagnostic kits were provided to technicians from provinces and counties.

##### Laboratory testing and score

The technicians in selected agencies were asked to perform the IHA and MHT tests according to the protocol of each method [12–14] and judge the results. The results were documented and reported to NIPD, China CDC within the given time. The total score of testing skills was 20, five points for IHA and MHT respectively and 10 points for snail identification. The detailed evaluation rules are provided in Additional file 1: Table S1.

#### Assessment on basic knowledge on schistosomiasis control

A questionnaire was designed by the professionals and experts on schistosomiasis prevention and control to assess the basic knowledge of medical staff responsible for schistosomiasis surveillance. The questionnaire was composed of three parts: knowledge of epidemiological and transmission characteristics of schistosomiasis japonica, diagnosis and treatment, case reporting and management. Each part consisted of five questions. The full questionnaire is provided in Additional file 2. Three professionals from CDC at provincial level and county level and two medical agencies at county level attended the assessment respectively. The assessment adopted the form of closed-book examination. The total score for the questionnaire was 10. One point would be deducted per error answer until the score was decreased to zero (Additional file 1: Table S1).

### Data management and statistical analysis

All data were transferred to Microsoft Excel software, version 2013 (Microsoft Office, CA, USA) for data compilation. The positive rate of serological test (number of antibody positives/number of serum samples examined) and the infection rate of cattle in different years and provinces, number of cases with stool examination positive, number of cattle with stool examination positive, and areas of newly detected snails in each county from 2005 to 2016 were calculated to analyze the epidemic situation. The results of assessments on diagnostic skills, snail identification and questionnaire survey were determined by the accuracy rate (number of correctly tested samples/number of total samples or number of correct answers/number of questions). 95% confidence intervals (CI) were calculated using standard formulae based on the binomial distribution. Microsoft Excel software, version 2013 (Microsoft Office, CA, USA) and the statistical software SPSS, version 23.0 (SPSS Inc., Chicago, USA) were used to analyze the epidemic trend based on descriptive analysis.

## Results

### Surveillance results of schistosomiasis

#### Surveillance results on population

From 2005 to 2016, a total of 3 569 509 serological tests were conducted for schistosomiasis screening and 24 978 blood samples were determined as antibody positives in the five PLADs (Table 1). The annual positive rate of serological tests was 0.56–0.90%, with a slight fluctuation from 2005 to 2010, and a sustained downward trend from 2011.

**Table 1:** Surveillance results of schistosomiasis on human beings in five PLADs during 2005–2016

Year	Serological tests			Stool examination	
	No. serum samples examined	No. antibody positives	Positive rate (%, 95 *CI*)	No. fecal examinations	No. stool positives
2005	428 800	3479	0.81 (0.78–0.84)	4565	44
2006	475 182	2681	0.56 (0.54–0.59)	4740	36
2007	476 201	3348	0.70 (0.68–0.73)	5876	27
2008	391 047	2647	0.68 (0.65–0.70)	3549	17
2009	339 583	3062	0.90 (0.87–0.93)	4145	21
2010	345 645	3057	0.88 (0.85–0.92)	3993	12
2011	240 121	1488	0.62 (0.59–0.65)	5167	12
2012	223 487	1332	0.60 (0.56–0.63)	3720	9
2013	221 765	1324	0.60 (0.57–0.63)	2570	14
2014	150 182	973	0.65 (0.61–0.69)	3520	10
2015	143 617	842	0.59 (0.55–0.63)	2563	8
2016	133 879	745	0.56 (0.52–0.60)	1589	11
Total	3 569 509	24 978	–	45 997	221

*No.* Number of. – means not applicable

Totally 45 997 stool examination were performed during the 12 years in five PLADs and 221 stool positives were detected (Table 1). Among the stool positives, Zhejiang, Shanghai and Fujian accounted for 87.33% (193/221), 11.31% (25/221) and 1.36% (3/221), respectively, while no stool positive cases were found in Guangdong or Guangxi. All stool positives were imported cases who got infection from other endemic areas.

#### Surveillance results on cattle

From 2005 to 2016, a total of 82 858 serological tests were conducted for surveillance on cattle in the five PLADs and 19 serological positives were found in Zhejiang Province, with 11 and 8 positive in 2014 and 2015, respectively (Table 2). Meanwhile, a total of 42 645 stool examination were performed, and no infected cattle was detected.

**Table 2:** Surveillance data of cattle in the five PLADs from 2005 to 2016

Year	No. serological tests	No. positive serological tests	No. stool examinations	No. positive stool examinations
2005	12 869	0	4812	0
2006	8846	0	4231	0
2007	8827	0	4027	0
2008	7525	0	2700	0
2009	7601	0	2593	0
2010	5730	0	2362	0
2011	6706	0	3109	0
2012	6068	0	2882	0
2013	5917	0	3031	0
2014	4878	11	3299	0
2015	4859	8	4368	0
2016	3032	0	5231	0
Total	82 858	19	42 645	0

*No.* Number of

#### Data on snail survey

Snail surveys were conducted in 126 837.75 hm^2^ of areas from 2005 to 2016 in the five PLADs. The area infested with living *Oncomelania hupensis* presented a descending trend, decreased from 112.70 hm^2^ in 2005 to 52.81 hm^2^ in 2016 (Table 3). Among the five PLADs, Guangdong maintained the status without snails’ infestation since 1992. The area of snail habitats in Zhejiang Province always accounted for the largest percentage, but presented a decrease trend from 80.72 hm^2^ in 2005 to 44.41 hm^2^ in 2016. However, no infected snail was found through dissection method in the five PLADs.

**Table 3:** Results of snail survey in the five PLADs during 2005–2016

Year	Areas conducted snail survey (hm^2^)	Areas with living snails (hm^2^)	Area of new snail habitats (hm^2^)	Area with recurrent snails (hm^2^)	Areas with mollusciciding (hm^2^)	Areas with environmental modification (hm^2^)
2005	13 181.83	112.70	1.26	^a^	261.58	9.06
2006	11 190.36	116.12	0.00	^a^	306.71	26.36
2007	11 059.95	126.90	4.66	^a^	239.83	23.34
2008	11 243.00	95.90	0.00	^a^	184.40	24.02
2009	10 735.92	100.26	0.00	^a^	110.07	15.12
2010	11 059.37	71.06	0.60	^a^	117.55	5.16
2011	10 046.77	58.52	0.82	51.74	118.06	4.64
2012	9161.18	79.15	0.61	61.52	320.61	2.77
2013	9326.08	62.52	0.43	35.64	123.77	16.33
2014	9441.76	48.46	1.18	28.90	123.29	3.43
2015	9833.01	47.52	0.43	32.60	103.23	3.88
2016	10 558.53	52.81	1.99	37.15	102.79	5.34
Total	126 837.75	–	11.98	247.55	2111.89	139.45

^a^means not available. – means not applicable

During the 12 years, 11.98 hm^2^ of new snail habitats (environments with no snails initially) were found in Zhejiang (6.53 hm^2^), Shanghai (4.19 hm^2^) and Guangxi (1.26 hm^2^). In addition, snail infestation reoccurred in 247.55 hm^2^ of former snail habitats since 2011, mainly distributed in Zhejiang (224.62 hm^2^), Guangxi (11.48 hm^2^), Fujian (10.73 hm^2^) and Shanghai (0.72 hm^2^).

Mollusciciding and extended mollusciciding were implemented among areas with snails. During the 12 years, mollusciciding were done with the area of 2111.89 hm^2^ and environmental modifications were done among some appropriate environments with the area of 139.45 hm^2^.

### Comprehensive assessments of surveillance capacity

#### Capacity for testing skills and snail detection

Totally 15 disease prevention and control agencies attended the assessment on testing skills and snails detection. For IHA, all professionals from the 15 agencies could perform and judge the results of five serum samples accurately, the accuracy rate was 100% (75/75). For MHT, the average accuracy rate was 89.3% (67/75, 95% *CI*: 82.2–96.5%), while eight wrong judgment results were all occurred in four CDC laboratories at county level.

**Fig. 2:**
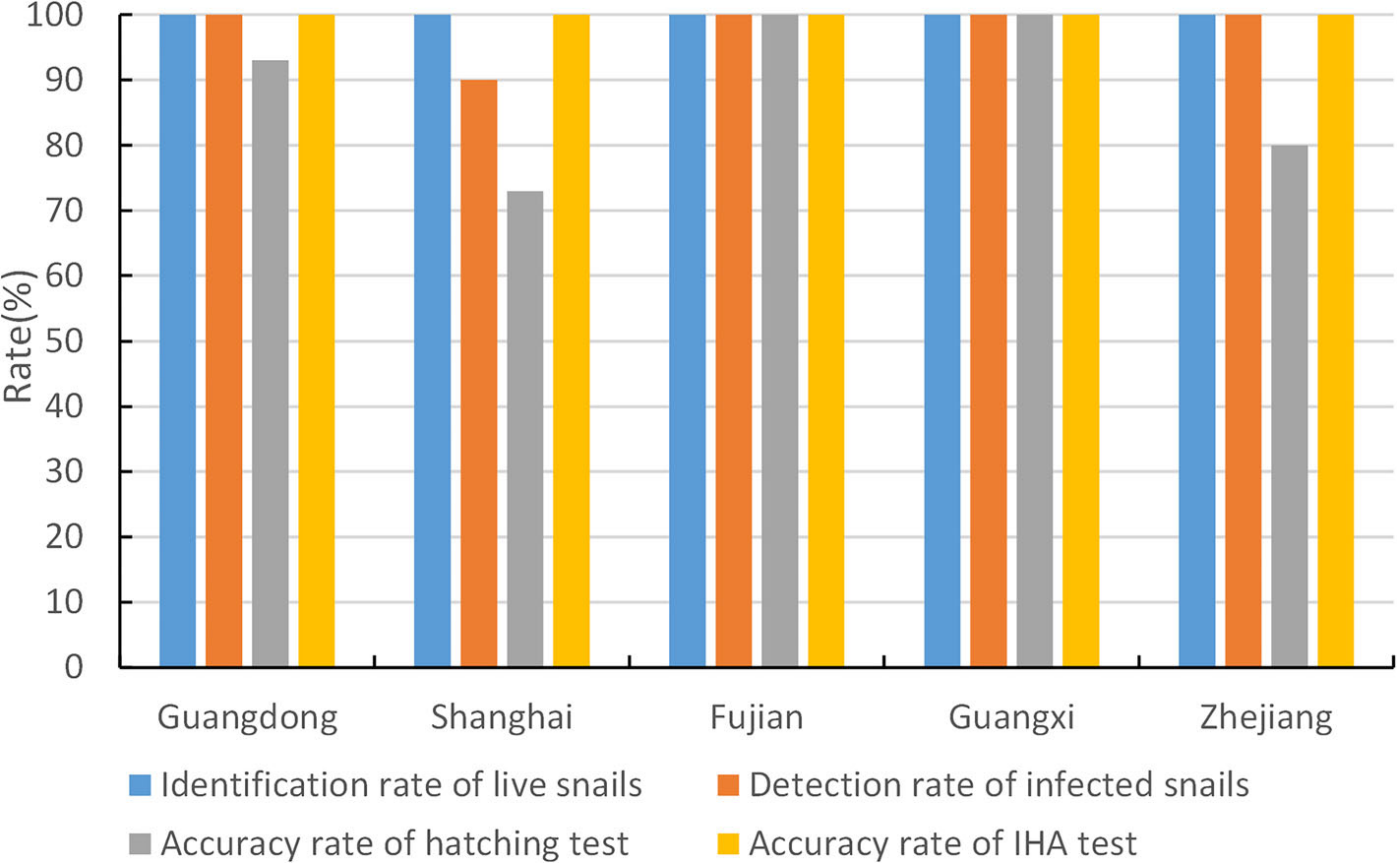
Evaluation results of schistosomiasis diagnosis and snail detection among fifteen institutions in the five PLADs

For snail identification, all agencies preformed excellent capacity to identify the snails’ living status with the accuracy rate of 100% (150/150) (Fig. 2). The average accuracy rate of identifying infection status of snails was 98.1% (103/105, 95% *CI*: 95.4–100.8%). And two wrong judgment results occurred in one CDC at county level.

#### Questionnaires survey on basic knowledge of schistosomiasis

Total of 108 medical staffs in 15 disease prevention and control agencies attended the questionnaire survey (Table 4). The results showed that the average accuracy rate of all respondents was 98.0% (529/540, 95% *CI*: 96.8–99.2%). Among five PLADs, respondents from Guangdong and Guangxi PLADs answered all questions correctly. The wrong-answered questions were mainly about knowledge on national comprehensive schistosomiasis control strategies and diagnosis of schistosomiasis.

**Table 4:** Scores of questionnaires on schistosomiasis control knowledge among professionals from 15 institutions in 5 PLADs and 10 counties

Province	Level of each agency	No. respondents	No. questions	No. correct answers	Accuracy rate (%)
Guangdong	Province	9	45	45	100.0
	Qingcheng	9	45	45	100.0
	Qujiang	9	45	45	100.0
	Subtotal	27	135	135	100.0
Shanghai	Municipality	3	15	15	100.0
	Pudong	9	45	45	100.0
	Songjiang	9	45	39	86.7
	Subtotal	18	90	84	93.3
Fujian	Province	3	15	14	93.3
	Xiapu	9	45	45	100.0
	Fuqing	9	45	44	97.8
	Subtotal	21	105	103	98.1
Guangxi	Autonomous region	3	15	15	100.0
	Jingxi	9	45	45	100.0
	Yizhou	9	45	45	100.0
	Subtotal	21	105	105	100.0
Zhejiang	Province	3	15	15	100.0
	Jiahsan	9	45	45	100.0
	Changshan	9	45	42	93.3
	Subtotal	21	105	102	97.2
Total		108	540	529	98.0

*No.* Number of

#### The total scores of the surveillance assessment

Based on the results of testing skills and snail detection and questionnaire surveys, except for Fujian scored 29 points, the scores of the other four PLADs were all 30 points (Table 5). In ten counties, the scores ranged from 26 to 30 points.

**Table 5:** Comprehensive assessment scores of schistosomiasis surveillance capabilities in five PLADs and ten counties

Province	Level of each agency	Diagnostic skills	Snail identification	Questionnaire survey	Total
Guangdong	Province	10	10	10	30
	Qingcheng	9	10	10	29
	Qujiang	10	10	10	30
Shanghai	Municipality	10	10	10	30
	Pudong	10	9	10	29
	Songjiang	6	10	4	20
Fujian	Province	10	10	9	29
	Xiapu	10	10	10	30
	Fuqing	10	10	9	29
Guangxi	Autonomous region	10	10	10	30
	Jingxi	10	10	10	30
	Yizhou	10	10	10	30
Zhejiang	Province	10	10	10	30
	Jiahsan	8	10	10	28
	Changshan	9	10	7	26

## Discussion

Although the definition of schistosomiasis elimination was announced by WHO in recent years and the debate of how to prove elimination is still going on, Guangdong (1985), Shanghai (1985), Fujian (1987), Guangxi (1988) and Zhejiang (1995) were announced that schistosomiasis was eliminated successively according to the criteria issued by Chinese government at that time. Then the five PLADs transferred to post-elimination surveillance with main tasks to find and eliminate local residual infectious sources and remaining snail habitats, and prevent the import of infectious sources and snails from other endemic areas [15–17]. Before our study, except one foci with two new cases reemerged in a farm of Guangdong Province in 1992 but rapidly was under controlled [17, 18], no new infections occurred in other PLADs [19].

In our study, no local infection in reservoir hosts and intermediate host was detected in the five PLADs during 2005–2016, proving the successful consolidation of schistosomiasis elimination. And to improve the surveillance effectiveness on schistosomiasis in China, the sensitivity and specificity of the diagnosis reagents were improved and validated [20]. The comprehensive assessment results showed that the staffs have mastered the basic knowledge of schistosomiasis prevention and presented good capacity for schistosomiasis detection. All of these are elementary components of a sensitive and rapid surveillance platform. However, we noticed that there were some samples misdiagnosed and wrong answers for questionnaire answered by staff at county level. Capacity building should be strengthened focusing on schistosomiasis control and diagnosis through continued training and practices to consolidate local achievements on schistosomiasis control.

However, risks of the re-emergence and resurgence of schistosomiasis still existed in the five PLADs through our study. Over the past 12 years, a total of 221 imported cases were found in the five PLADs, mainly in Zhejiang Province, neighbored with Jiangxi and Anhui provinces where the transmission of schistosomiasis is still going on. Studies showed that most of the imported cases in Zhejiang Province are farmers, migrant workers and merchants from schistosomiasis endemic areas, primarily from Anhui, Jiangxi, Hubei [21–23]. In recent years, owing to urbanization and economic development, the number of the floating population from domestic epidemic areas has an increasing trend. At the same time, with the escalation of international trade and entry-exit personnels, the risk of importing cases from abroad infected with *S. haematotium* or *S. mansoni* is also increasing [24–26]. In view of this situation, on the one hand, supervision and treatment should be strengthened for imported definite cases to eliminate the transmission potential; on the other hand, health education should be promoted for the floating population from schistosomiasis endemic areas to spread the knowledge of schistosomiasis prevention and control [27].

The surveillance data proved that the area of snail habitats kept a low level during 2005–2016, compared with 95 900.70 hm^2^ reported by Wu [17]. The remaining snail habitats mainly distributed in the places that the ecological environments are complicated or water level is unstable, where molluscaciding approach doesn’t work well. In addition, several articles published also presented the evidence that the rebound or spread of snails habitats were occurred in Shanghai, Fujian, Guangxi and Zhejiang [17, 28–30]. It is worth noting that the connection of water systems or the transplanting of seedlings and aquatic plants from the snail habitat areas may also lead to the possibility of snail importation and spread [31, 32]. The development of new snail habitats and snails reappeared in former snail habitats in four provinces except Guangdong Province, providing evidence that eliminating *Oncomelania* snails completely was quite difficult. Although Guangdong Province kept the achievement with no *Oncomelania* snails detected, a new challenge for Guangdong Province is the invasion and spread of *Biomphalaria straminea* [33].

Considering the potential risks of schistosomiasis still existed in the five PLADs, snail control through environmental modification and surveillance focused on eliminating remaining snails and preventing imported infection sources should be continued and strengthened, to prevent the re-emergence of schistosomiasis, and consolidate the achievements of schistosomiasis elimination. Risk assessment should be conducted timely if there were large water conservancy projects or importing plants or animals from endemic areas, etc. [34, 35]. Specifically, the monitoring of the environments where snails infested previously or connected with snail habitats should be strengthened through multi ways [10, 36]. Meanwhile, the floating people and livestock from the areas where the transmission of schistosomiasis has not been interrupted or the epidemic situation of schistosomiasis is recovering should be inspected emphatically, and the patients should be treated in time if they are found [37–39].

Several limitations of this study should be noted. One is that data collection on surveillance results is based on the retrospective data collection on human, cattle and snails, some detailed information especially personal information on human with serum positive couldn’t be available. And some deep analysis on human couldn’t be implemented. The other one is only two counties were selected from each PLADs to evaluate the surveillance capacities, and there were 112 counties with schistosomiasis endemic in this five PLADs, so there may be a selection bias in the results of assessments on the surveillance capacities.

## Conclusions

Elimination of schistosomiasis was consolidated successfully in five PLADs of P. R. China due to effective and strong post-elimination surveillance. Being a zoonotic parasitic diseases, challenges still exist to maintain the achievements as imported cases and snail habitats were detected during 2005–2016. Continuous surveillance should be strengthened through capacity building for staff responsible for schistosomiasis surveillance, providing adequate funding and resources.

## Supplementary information

**Additional file 1: Table S1.** Detailed grading rules for the assessment on surveillance capacity in the five provinces.

**Additional file 2.** The questionnaire on basic knowledge of schistosomiasis control.

## Data Availability

All data generated or analyzed during this study are kept confidential by NIPD, China CDC. The datasets are available from the corresponding author on a reasonable request.

## Abbreviations

CDC: Center for Disease Control
CI: Confidence intervals
MHT: Miracidia Hatching technique
IHA: Indirect hemagglutination assay
NIPD: National Institute of Parasitic Diseases
P. R. China: People’s Republic of China
WHO: World Health Organization
