# Supplementary material for: Surveillance on schistosomiasis in five provincial-level administrative divisions of the People’s Republic of China in the post-elimination era
Source: Infect Dis Poverty. 2020 Oct 1;9:136. doi: 10.1186/s40249-020-00758-4 (PMC7528395; doi:10.1186/s40249-020-00758-4)
Supplement: Supplementary file 1 — Additional file 1: Table S1. Detailed grading rules for the assessment on surveillance capacity in the five provinces. [file 40249_2020_758_MOESM1_ESM.docx]

Additional file 1: Table S1. Detailed grading rules for the assessment on surveillance capacity in the five provinces.

| Evaluation content | Methods | Standard for evaluation | Score |
| --- | --- | --- | --- |
| Diagnostic skills | The indirect hameagglutination assay (IHA) and the miracidia hatching technique (HT) | Five serum samples were tested, all results were correctly judged, +5 points; for each error, -1 point, and five stool samples were hatched, all results were correctly judged, +5 points; for each error, -1 point. | 10 |
| Snail identification | Snails dissection and microscopic method | The live state of ten snail samples were identified, with all snails correctly identified, +5 points; for each error, -0.5 points. The infection state of ten snail samples were identified, with all snails correctly identified, +5 points; for each error, -0.5 points. | 10 |
| Basic knowledge on schistosomiasis control | Three staff from each selected organization attended the questionnaire survey | Each person answered 5 questions according to his/her major, with all 15 questions correctly answered, +10 points; for each error, -1 points until zero. | 10 |
| Total |  |  | 30 |
