# Supplementary material for: Surveillance on schistosomiasis in five provincial-level administrative divisions of the People’s Republic of China in the post-elimination era
Source: Infect Dis Poverty. 2020 Oct 1;9:136. doi: 10.1186/s40249-020-00758-4 (PMC7528395; doi:10.1186/s40249-020-00758-4)
Supplement: Supplementary file 2 — Additional file 2. The questionnaire on basic knowledge of schistosomiasis control. [file 40249_2020_758_MOESM2_ESM.docx]

Additional file 2: The questionnaire on basic knowledge of schistosomiasis control.

Part one. Case reporting and management

1. The Law of the People’s Republic of China on the Prevention and Control of Infectious Diseases was revised and adopted at the 11th meeting of the Standing Committee of the Tenth National People’s Congress on August 28, 2004. Schistosomiasis was classified as ( ) infectious diseases.

A. Class A B. Class B C. Class C D. Non-infective disease

2. Medical agencies and disease prevention and control institutions at all levels should report to the health administrative department of the local county government as soon as possible within ( ) when they discover a sudden outbreak of schistosomiasis or that a possible outbreak of schistosomiasis.

A. 1 hour B. 2 hours C. 3 hours D. 4 hours

3. When schistosomiasis cases are found, medical agencies at all levels should fill in the infectious disease report card within ( ) after diagnosis of the cases, and directly report through the infectious disease report information management system of Chinese Center for Disease Control and Prevention.

A. 1 hour B. 4 hours C. 24 hours D. 72 hours

4. Which of the following situations is not a sudden outbreak of schistosomiasis ( )

A. In the area of schistosomiasis transmission control, there were more than 5 cases of acute schistosomiasis within 2 weeks in the same administrative village;

B. In the area of schistosomiasis transmission interruption, local cases, infected livestock and infected snails were found;

C. New snail habitats were found in the area of schistosomiasis transmission interrupted;

D. In the non-endemic area where schistosomiasis has never been found in history, local cases were found.

5. In endemic areas, the susceptible season of schistosomiasis is ( )

A. From January to February B. From April to October

C. From November to December D. Four seasons of the year

Part two. Schistosomiasis diagnosis and treatment

1. Which of the following is not a common method for serological diagnosis of schistosomiasis ( )

A. Dot Immunogold Filtration Assay, DIGFA

B. Indirect Hemagglutination Assay, IHA

C. Complement Fixation Test, CFT

D. Enzyme-Linked Immunosorbent Assay, ELISA

2. The diagnosis of schistosomiasis cases is based on the ( )

A. Immunologic test B. Etiological examination

C. Ultrasound examination D. Magnetic Resonance Imaging, MRI

3. The first choice for treatment of schistosomiasis is ( )

A. Albendazole B. Artemisinin C. Decoction of areca and pumpkin seeds

D. Praziquantel

4. After a person infection with *Schistosoma japonicum*, the earliest time to find the eggs in the feces is about ( )

A. 15 days B. 35 days C. 60 days D. 90 days

5. According to the time of onset, symptoms and signs, schistosomiasis can be divided into ( )

A. Early stage, middle stage and late stage B. Acute, chronic and advanced stage

C. Suspected, clinical and confirmed D. All three above

Part three. Comprehensive knowledge

1. After 2006, the focus of the national comprehensive schistosomiasis control strategies turned to ( )

A. Controlling the source of infection

B. Eliminating snails

C. Protecting the susceptible people

D. Replacing cattle with machines

2. The infective route of schistosomiasis is ( )

A. Eating raw freshwater fish and shrimp

B. Eating raw aquatic plants

C. Skin infection through contact with infected water

D. Skin infection through contact with soil

3. The intermediate host of *Schistosoma japonicum* is ( )

A. *Oncomelania hupehensis* B. *Planorbis planorbis*

C. *Pomacea canaliculata* D. *Parafossarulus striatulus*

4. The latest version of the criteria of schistosomiasis control or elimination has been implemented since ( )

A. May 1, 2014 B. May 1, 2005 C. January 1, 2016 D. January 1, 2010

5. For schistosomiasis surveillance in the areas where the transmission of schistosomiasis has been interrupted, the people who need to be monitored in particular are ( )

A. Local population B. Floating population

C. Floating population from endemic areas D. European tourists
